# Marmesin and Marmelosin Interact with the Heparan Sulfatase-2 Active Site: Potential Mechanism for Phytochemicals from Bael Fruit Extract as Antitumor Therapeutics

**DOI:** 10.1155/2023/9982194

**Published:** 2023-01-05

**Authors:** C. Hemakumar, B. S. Ravindranath, G. A. Ravishankar, D. C. Ramirez, S. V. Kiran

**Affiliations:** ^1^Department of Biotechnology, Dayananda Sagar College of Engineering, Shavige Malleshwara Hills, Kumaraswamy Layout, Bangalore 560111, Karnataka, India; ^2^Department of Biotechnology, Manipal Institute of Technology, Manipal Academy of Higher Education, Eshwar Nagar, Manipal, 576104 Karnataka, India; ^3^Laboratory of Experimental and Translational Medicine, IMIBIO-SL, CCT-San Luis (CONICET), National University of San Luis, 5700 San Luis, San Luis, Argentina

## Abstract

Human heparan sulfatase-2 (HSULF-2) is an oncoprotein overexpressed in the surface of all types of tumor cells and its activity plays a critical role in cancer survival and progression. Our previous studies have shown that bael fruit extract, containing marmesin and marmelosin, inhibits the HSULF-2 activity and kills breast tumor cells, but the mechanism of these processes remains fairly known mainly because the HSULF-2's 3D structure is partially known. Herein, we aimed at providing an *in silico* molecular mechanism of the inhibition of human HSULF-2 by phytochemicals from bael fruit extract. Pharmacokinetic parameters of the main phytochemicals contained in the bael fruit extract, sequence-based 3D structure of human HSULF-2, and the interaction of bael fruit's phytochemicals with the enzyme active site was modeled, evaluated, and verified. Docking studies revealed marmesin and marmelosin as potential inhibitors with binding score -8.5 and -7.7 Kcal/mol; these results were validated using molecular dynamics simulations, which exhibited higher stability of the protein-ligand complexes. Taking together, with our earlier *in vitro* data, our computational analyses suggest that marmesin and marmelosin interact at the active site of HSULF-2 providing a potential mechanism for its inhibition and consequent antitumor activity by phytochemicals contained in the bael fruit extract.

## 1. Introduction

Cancer is one of the most life-threatening diseases affecting both males and females, and there are about 19.3 million new cases reported by the GLOBOCAN in the year 2020 (GLOBOCAN 2020—International Agency for Research on Cancer (IARC)). These data suggest an urgent need for pioneer research on natural products, particularly the search for phytochemicals to enhance the development of effective and safe antitumoral drugs.

Currently, most of the drugs used to treat cancer are either phytochemicals/nutraceuticals or synthetic derivatives. Indeed, 90 out of 120 antitumoral drugs available are phytochemicals [1]. Several studies on the mechanism of action of these phytochemicals suggest that they modulate cell signaling, cell cycle, oxidative stress response, and inflammation [2]. Among the numerous dietary nutraceuticals, bael fruit (*Aegle marmelos* (L.) Correa) has gained considerable attention due to its antioxidant, anti-inflammatory, antitumoral, and antidiabetic properties [3–5]. These properties of the bael fruit are suggested to be due to its phytochemicals, including tannins, skimmianine, essential oils like caryophyllene, cineole, citral, cuminaldehyde, citronella, p-cymene, d-limonene, and eugenol sterols or triterpenoids, including lupeol, *β*- and *γ*-sitosterol, *α*- and *β*-amyrin, flavonoids like rutin and coumarins, including aegeline, marmesin, umbelliferone, marmelosin, marmelin, leucoanthocyanins, and anthocyanins [6]. Among these phytochemicals, marmelosin and marmelin have attracted much attention as bioactive compounds with antitumoral and anti-inflammatory properties [5, 7]. For instance, it has been reported that marmelin activates programmed tumor-cell death through induction of tumor necrosis factor-*α* signaling in breast cancer cells [8].

Two isoforms of the human heparan sulfate 6-O-endosulfatases (HSULF, HSulfs, and EC 3.1.6.14) have been described, HSULF-1 and HSULF-2 [9]. They belong to the heparan sulfatase 6-O-endosulfatase family, which were first discovered in the early 2000s in quail embryos [10, 11]. These enzymes catalyze the removal of the 6-O-sulfate group of the glucosamine residues of heparan sulfate [11–13]. These isoforms are structurally similar, but they perform different pathophysiological functions [12, 14–16]. HSULF-1 is a tumor suppressor protein [17], whereas the role of HSULF-2 in pathophysiology remains partially known [18]. However, previous studies have shown that HSULF-2 is upregulated and promotes hepatic [17, 19, 20], renal [21], colorectal [22], pancreatic [23–25], ovarian, breast [26, 27], and lung [28] cancer. Although there are some pieces of evidence about the role of HSULF-2 in cancer, its role in other noncommunicable diseases is partially known [29]. Based on these studies, HSULF-2 activity is an important oncoprotein in cancer progression and thus an important therapeutic target [29–31]. However, although the primary structure of this oncoprotein is known [32], computational modeling of the 3D structure of human HSULF-2 is still unavailable. The 2,4-disulfophenyl-*N*-*tert*-butyl nitrone is a synthetic compound that can inhibit the enzymatic activity of heparan sulfatase-2 and kills hepatocellular carcinoma cell lines [33]. However, research on phytochemical compounds that can inhibit the enzymatic activity of HSULF-2 is rare [34, 35]. Recently, we have shown that an extract from bael fruit inhibits the activity of HSULF-2 and kills the MCF-7 breast cancer cell line [35]. However, the mechanism for this inhibition is unclear.

Nowadays, *in silico* screening has become an important tool in discovering varieties of drugs wherein ligands, otherwise called hit compounds, are accounted for. The *in silico* analyses toolbox help identify bioactive compounds that could be potential hit compounds for the protein target, as well as their pharmacokinetics. Deep knowledge of the inhibition of the HSULF-2 activity by isolated phytochemicals is critical to avoid off-target effects in case they are moved into clinical trials for cancer therapy.

Herein, we aimed at providing robust evidence regarding the role of the human *hsulf-2* gene in noncommunicable human diseases. We also predicted the sequence-based 3D structure of this oncoprotein by using the Iterative Threading ASSEmbly Refinement (I-TASSER) method, which gives structure-based functional annotation. By combining all docking studies, nonbonding protein interactions, and pharmacokinetics, we found a couple of five-hit compounds, including marmesin and marmelosin. Our results may help find phytochemicals that can serve as a structural platform for the synthesis of structural analogs for the inhibition of HSULF-2 activity and thus be effective and safe antitumor drugs.

## 2. Materials and Methods

### 2.1. Gene-Disease Functional Network Prediction for Human HSULF-2

Based on the known guide genes, we can predict the new candidate genes involved in the disease of interest using HumanNet. HumanNet 2 was employed to predict the functional network using the guide *hsulf-2* gene. HumanNet-FN network was opted for prediction and further selected network-based disease-gene prediction to submit the guide gene. The queried *hsulf-2* guide gene helped in predicting the candidate genes possibly involved in the disease. Later, we selected network-based disease-gene annotation prediction to predict the linked diseases. The candidate genes are ranked based on the summation of the edge scores obtained directly linked to the guide gene. The genes with high-rank scores would be candidate gene members involved in the disease [36]. HumanNet also provides predefined guide genes based on multiple databases, including DSigDB, DisGeNET, Diseases, DOAF, HPO, and GWAS catalog databases.

### 2.2. HSULF-2 Sequence Data Retrieval and Analysis

The protein sequence with the sequence id: Q8IWU5 (HSULF-2 _HUMAN) was obtained in FASTA format from the NCBI database (http://www.ncbi.nlm.nih.gov/protein/). The HSULF-2 protein sequence information was gathered through UniProt (https://www.uniprot.org/) high quality and freely accessible protein database. The information related to family superfamilies of Q8IWU5 (HSULF-2 _HUMAN) was retrieved using the InterProScan tool [37]. Domains of the protein were predicted using SMART [38] and CDD [39].

### 2.3. Template Search and HSULF-2 Sequence Alignments for Secondary Structure Prediction

**Figure 1:**
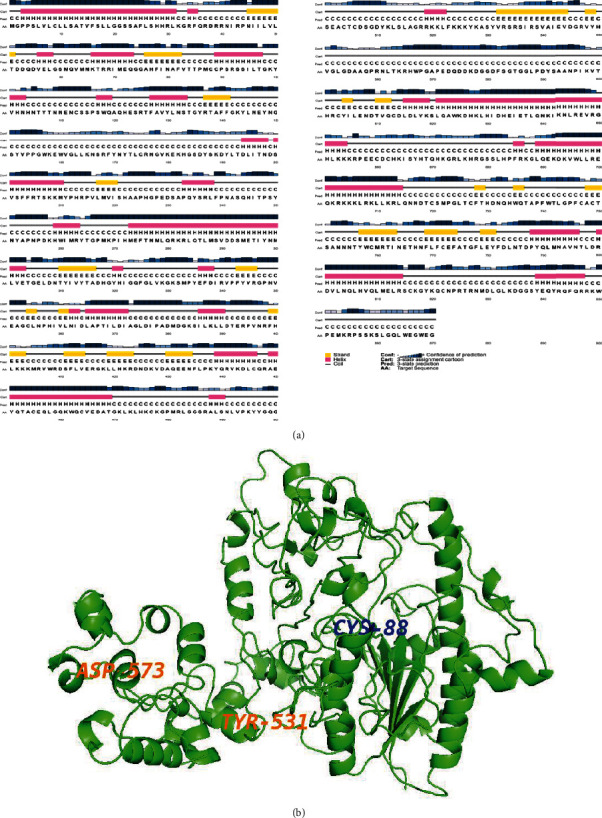
Predicted secondary structure (PSIPRED) and 3D structure of human HSULF-2 based on the amino acidic sequence. (a) The prediction confidence is shown according to the color intensity. The yellow region depicts strands, the pink region represents alpha-helix, and grey line indicates coils. (b) The structure (residues 41-683) shown in green is considered for further structural validation. The somatic mutations (ASP^573^ and TYR^531^) leading to breast cancer are labeled in orange. The active site (CYS^88^ in blue) and binding site residues are falling well within the considered modeled protein structure which has been validated using PROCHECK, ProSA-web, and Verify 3D.

PSI-BLAST (http://blast.ncbi.nlm.nih.gov/Blast.cgi) [40] was employed to identify the homologous structure for the target protein sequence against Protein Data Bank (PDB) [41]. The multiple sequence alignment was performed to validate sequences similar to the structure. The sequences from the previous template search were aligned. The target protein (Q8IWU5) and the selected template Evalue = 0 and percent identity > 40. Selected templates and target protein sequences were aligned using PRALINE with default parameters [42]. The Secondary structure prediction was performed using the PSIPRED server (Figure 1) [43].

### 2.4. 3D HSULF-2 Structure Modeling and Refinement

I-TASSER (Iterative Threading ASSEmbly Refinement) approach was used to predict the 3D structure of the target protein and also to perform structure-based functional annotation [44]. Structural templates were identified for the protein (Q8IWU5) using multiple threading approaches and functions were derived by rethreading the developed models through the protein function database. The ModRefiner tool (https://zhanglab.ccmb.med.umich.edu/ModRefiner/) was implemented for the refinement of protein structure. It is an algorithm helpful in performing atom-level structural refinement with high resolution. The refinement starts from the C-alpha trace by either considering the main-chain or full-atomic models. ModRefiner's overall purpose [44] is to model the basic forms of models nearer to their native state, based on their hydrogen-bond formation, topology, and side-chain placement. To evaluate the residues in the allowed region and the disallowed region of the Ramachandran plot, the tool employed was PROCHECK [45, 46]. The HSULF-2 3D structure quality was verified using the PROCHECK (https://www.ebi.ac.uk/thornton-srv/software/PROCHECK/). Verify 3D [47] and ProSA-web tools were adopted to assess the quality of modeled structure.

### 2.5. HSULF-2 Active Site Prediction in its 3D Structure

DoGSiteScorer online server allowed the assessment of the structure-based active site for the HSULF-2 [48]. It helps in the automatic and accurate prediction of the HSULF-2 target site for docking in the 3D protein structure. The identified ligand binding sites in the modeled protein structure (Q8IWU5) were 05 using DoGSiteScorer. The 3D structure of the protein obtained through the I-TASSER was extracted in the pdbqt file extension using AutoDock tools (http://vina.scripps.edu/) [48].

### 2.6. ADMET Prediction for Bael Fruit's Phytochemicals

Predicting absorption, distribution, metabolism, excretion, and toxicity (ADMET) properties is a significant step in ranking the chemical molecules as drug compounds in computer-aided drug designing (CADD). SwissADME is a web-based free access tool (http://www.swissadme.ch) [49] to predict the ADMET properties of the bael fruit's phytochemicals for user-friendly usage. It includes proficient methods like iLOGP and BOILED-Egg. It is significant due to its specific features like multiple input methods, the ability to compute a list of phytochemicals in a single run, and capability of displaying and downloading results of single molecule or share results per individual molecule or spontaneously all the predicted results in specific formats with interactive graphs. It is also integrated well with other workspaces to perform multiple functions like virtual screening, molecular modeling, target prediction, molecular docking, and molecular mechanics using several computational tools, such as SwissSimilarity, SwissDrugDesign, SwissTargetPrediction, and SwissDock.

### 2.7. *In Silico* Docking Studies for Selected Bael Fruit's Phytochemicals at the HSULF-2 Active Site

Molecular interaction mode analysis is crucial in the structure-based discovery of efficient drugs against different diseases for protein-ligand complexes. The selected docked bael fruit's phytochemicals (lupeol, l-ascorbic acid, marmesin, niacin, psoralen, marmelosin, and thiamine) were screened with the corresponding binding sites at the active site of HSULF-2. AutoDock Vina v4.2 [50] was used to initiate the docking procedure of the inhibitors (bael fruit's phytochemicals) with the protein target (HSULF-2). The modeled HSULF-2 protein was refined using ModRefiner and further analyzed based on PROCHECK, Verify 3D, and ProSA-web. Furthermore, it was subjected to docking by making the HSULF-2 as a macromolecule and saving it in pdbqt format. Similarly, the ligands were prepared by energy minimizing using the Open Babel tool [51] available in GUI-based PyRx [52], the default energy minimization parameters were applied, and the ligand was saved in pdbqt format. The grid box coordinates were defined as *x* = −28.70, *y* = −185.00, and *z* = −67.41, and the grid box dimensions of *x*, *y*, and *z* were set to 26.61, 26.49, and 25.37, respectively. The best HSULF-2-phytochemical complex with the lowest docking energy was selected to generate the 2D plot and postdock analysis based on MD simulations and free energy calculations. The information related to 2D phytochemical-HSULF-2 interactions was obtained using the Discovery Studio Visualizer. It reveals the interactions mediated by hydrogen bonds and hydrophobic-mediated interactions and van der Waals forces.

### 2.8. Molecular dynamics Simulation Study of the HSULF-2-Marmesin/-Marmelosin Complex

The protein-ligand complexes (HSULF-2-marmesin and HSULF-2-marmelosin complexes) with the best binding scores were selected as the starting coordinates to execute MD simulation of all atom for 100 ns using GROMOS96 54a7 force-field in GROMACS-2019 software to analyze the protein-ligand complex stability of the HSULF-2-phytochemical complexes [53]. The topology parameters for compounds marmesin and marmelosin were generated using a web-based PRODRG2 Server, and the same was incorporated with the protein topology to form a complex system. The generated complex system was fully solvated using the Simple Point Charge (SPC) water model in the dodecahedron box to form a simulated aqueous environment, and neutralization was performed by adding the sodium ions as stated in the earlier reports [54]. Based on the steepest descent method, an energy minimization step was performed to remove the potential steric clashes in the complex system at 2,000 steps. The position restraints were adopted for both complex systems, and temperature (at 300 K) and pressure (at 1.0 bar) balancing was performed for 100,000 ps. Finally, the system was subjected to MD simulation production runs for 100 ns by maintaining temperature (330 K) and pressure (1.0 bar). Subsequent trajectories were further analyzed using the inbuilt utilities of GROMACS to estimate the Root Mean Square Deviation (RMSD) and the Root Mean Square Fluctuation (RMSF) of the individual amino acid positions with the reference structures to analyze the structural flexibilities through the 100 ns simulation time.

## 3. Results

### 3.1. Disease-Gene Functional Network Analysis for the *Hsulf-2* Gene

Based on HumanNet 2 analysis, we were able to predict the 119 candidate genes interacting with the *hsulf-2* gene with the threshold of 5.10, and we limited our search to the top ten candidate genes with the distance (with threshold value 4) as shown in the network (Supplementary Figure 1A). Further, based on the network-based disease-gene annotation prediction, we annotated the thirty-three diseases from multiple databases DisGeNET, Diseases, DOAF, and GOBP, as shown in Supplementary Table 1. The diseases annotated primarily included multiple cancers, carcinomas, viral diseases, and diseases linked to multiple organelles. STRING network illustrates the proteins interacting with HSULF-2 involved in multiple disease networks (Supplementary Figure 1B).

### 3.2. Data Retrieval and Sequence Alignment of HSULF-2 Primary Structure

The amino acid sequence length of SULF_2 HUMAN was 870 aa. InterProScan revealed that the protein HSULF-2 belongs to the sulfatase family. The domains identified using the InterProScan tool were alkaline phosphatases-like core domain superfamily and Extracellular_sulfatase_C. The PSI-BLAST hit gave the homologous structure identified for the target protein sequence and it was 36%. The templates and the target protein sequence aligned showed identity when using PRALINE.

### 3.3. Modeling and Refinement of the 3D Structure of HSULF-2

In the current study, by using I-TASSER, we identified ten 3D-structure models of the protein HSULF-2. The first model Figure 1(a) was selected as the best one based on RMSD values, TM-score, and C-score after evaluating all the predicted models. The HSULF-2 protein has three domains, and hence, it is observed that there are divergent corelations between C-score and the overall model qualities. The best model was evaluated for the residues in the allowed and disallowed regions using PROCHECK (Figure 1(b)). The percentage of residues in the allowed and disallowed regions was 11.5% and 1.8%, respectively. The functions of the protein identified were arylsulfatase activity, calcium ion binding, glycosaminoglycan binding, and *N*-acetylglucosamine-6-sulfatase activity. The best model 4ug4_A was with RMSD 1.30 and TM-score 0.525 and was refined using the ModRefiner tool. Figure 1(b) shows the refined model of the modeled structure. The quality of the model that was refined was reevaluated and had a TM − score = 0.9980.

### 3.4. Model Validation of the 3D Structure of HSULF-2

**Figure 2:**
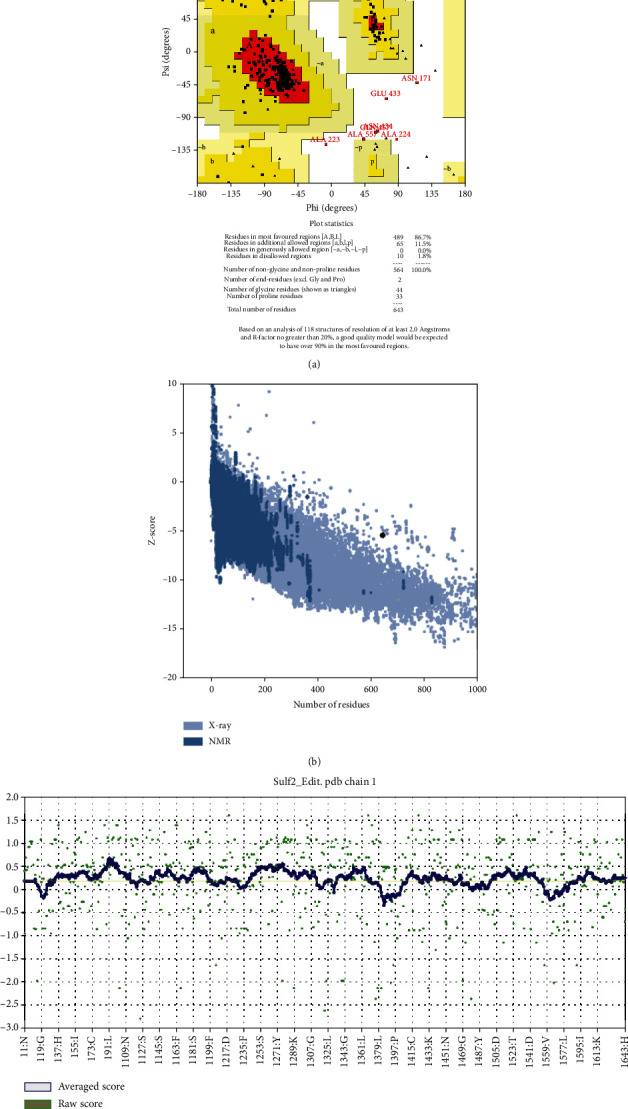
Validation and verification of the computational obtained 3D structure of HSULF-2. (a) Ramachandran plot with 86.7% residues falling in the most favored region and 11.5% residues in additional allowed regions. (b) ProSA-web-based validation with Z-score -5.48. (c) Verify 3D-based protein validation with 69.36% quality factor.

The results from PROCHECK were satisfying as the 86.7% residues were in the most favorable region, 11.5% residues were in the allowed region, and 1.8% residues in the disallowed region were of the Ramachandran plot (Figure 2(a)). Further, ProSA-web was used to validate the model, which showed a Z-score of -5.48, indicating the selected model's reliability (Figure 2(b)). Verify 3D results indicated 69.36% of the residues with an average 3D–1D score of ≥0.2, demonstrating a good correlation between the primary sequence and the 3D model (Figure 2(c)).

### 3.5. ADMET Analysis of Bael Fruit's Phytochemicals Interacting with HSFULF-2 Active Site

The query molecules (six) were submitted to SwissADME as a list of SMILES to predict the ADMET properties. The output includes major classifications like physicochemical properties, lipophilicity, water solubility, pharmacokinetics, drug-likeness, and medicinal chemistry. Physicochemical properties such as molecular weight, H-bond donors, H-bond acceptors, and TPSA score were predicted. In the lipophilicity section, Log *P* was recorded and water solubility section helped predict Log *S* (ESOL) and classify pharmacokinetics properties, including gastrointestinal (GI) absorption and blood-brain barrier (BBB) permeability. Under drug-likeness, Lipinski's rule of five was noted, and in the medicinal chemistry section, PAINS scores were recorded. All the individual results have been illustrated in Table 1. The results indicated the drug ability of the selected molecules based on the expected specific cut-off.

### 3.6. Molecular Docking Analysis of the HSULF-2 with Marmesin and Marmelosin

**Figure 3:**
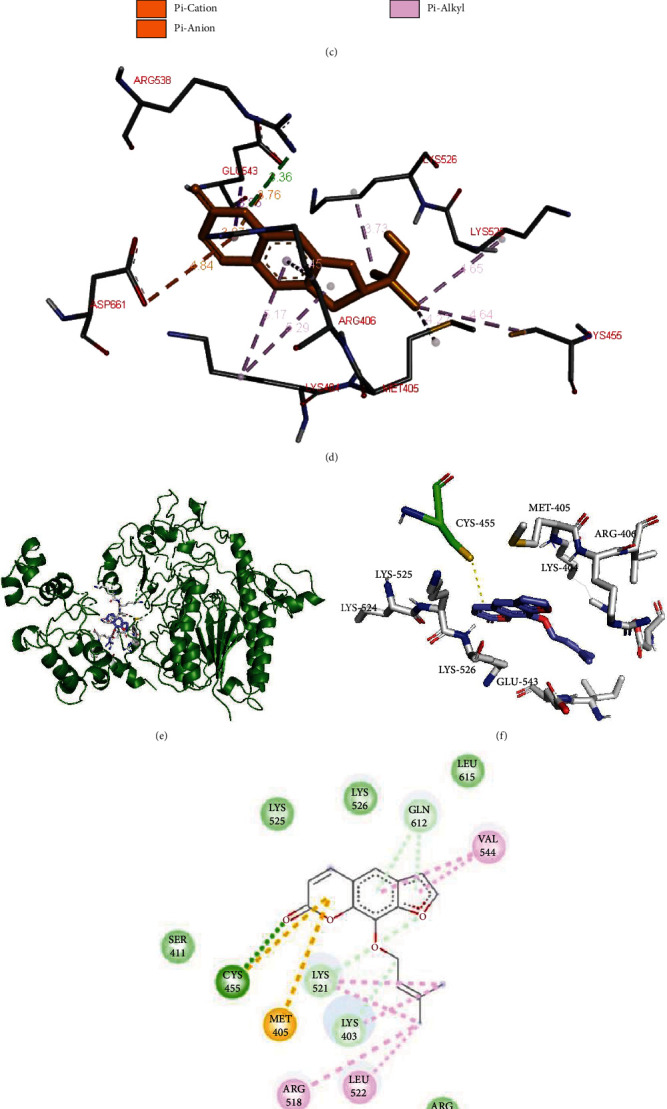
Interaction of marmesin and marmelosin at the active site of human HSULF-2. (a) Marmesin interacting in the binding site of HSULF-2 protein. (b) Depiction of binding site residues interacting with marmesin. (c) 2D plot of marmesin interaction with H-bonds and hydrophobic interactions. (d) Depiction of marmesin interaction forming H-bonds with binding site residues. (e) Marmelosin-HSULF-2 complex. (f) Marmelosin interaction with binding site residues. (g) 2D plot of marmelosin interaction forming multiple types of bonds. (h) Depiction of hydrophobic and hydrophilic regions.

To perform the analysis of the binding affinity of the selected molecules for their binding potential through the means of docking analysis, the docking procedure was conducted. The obtained docking results specified high-binding affinity between protein-ligand complexes; the same were selected based on binding scores at upper and at lower RMSD scores as zero Figure 3. The careful visualization (PyMol and Discovery studio) [55, 56] of the marmesin-HSULF-2 complex (Figures 3(a)–3(d)) resulted in higher binding affinity with a binding energy score of -8.5 Kcal/mol was stabilized with significant bonds with HSULF-2 in which two bonds are conventional H-bonds ((ARG^538^ (3.4 Å) and LYS^526^ (2.1 Å)), three pi-cation/pi-anion bonds (ARG^406^, GLU^543^, and ASP^661^), and seven alkyl/pi-alkyl bonds (LYS^404^, LYS^525^, MET^405^, and CYS^455^) (see Table 2). Marmelosin-HSULF-2-docked complex analysis (Figures 3(e)–3(h)) revealed significant interactions with binding energy score -7.7 Kcal/mol by forming one conventional H-bond (CYS^455^(2.5 Å)), three alkyl bonds (ARG^518^, LEU^522^, and VAL^544^), three carbon hydrogen bonds (LYS^521^, LYS^403^, and GLN^612^), one *π*-sulfur bond (MET^405^) and van der Waals interactions with five residues surrounding the binding pocket (ARG^519^, SER^411^, LEU^525^, LEU^526^, and LEU^615^). The above-mentioned docking results with significant interactions and binding scores are illustrated in Figures 3(a)–3(h).

To validate the results obtained from our docking analysis, the best scoring docked complexes were further considered for molecular dynamics (MD) simulation analysis.

### 3.7. Molecular Dynamics Simulation Analysis of the Interaction between Bael Fruit's Phytochemicals and the HSULF-2 Active Site

**Figure 4:**
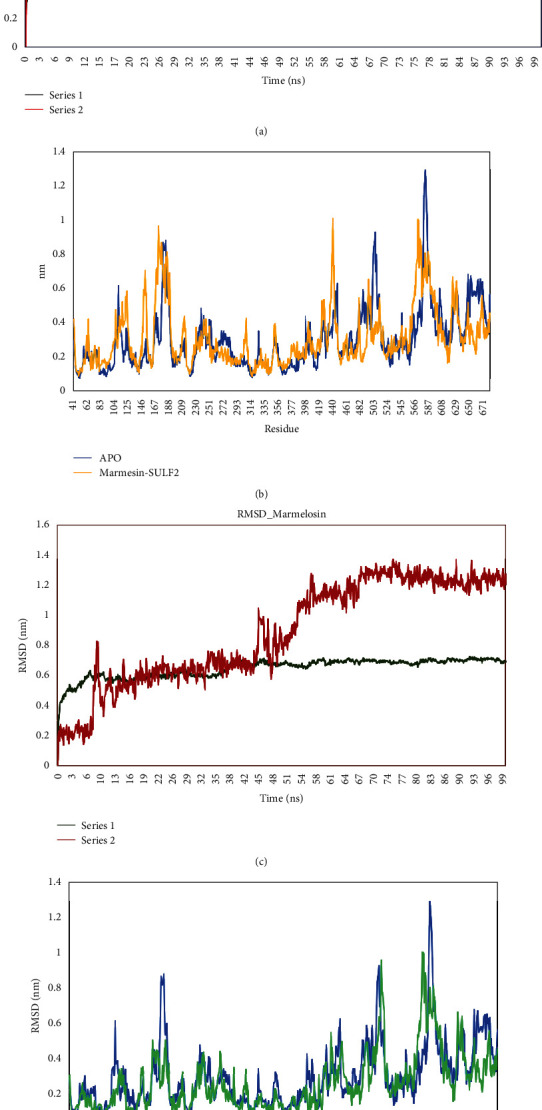
MD simulation of HSULF-2 interacting with marmesin and marmelosin. (a) RMSD plot of marmesin- (red) HSULF-2 (green) complex. (b) Root mean square fluctuation (RMSF) plot of marmesin-HSULF-2 complex (representing higher confirmation). (c) RMSD plot of marmelosin- (maroon) HSULF-2 (green) complex at varied time frame (0-100 ns). (d) RMSF plot of APO (green) and marmelosin-HSULF-2 (exhibiting comparatively superior confirmation).

The protein (HSULF-2) and docked complex (marmesin-HSULF-2) were subjected to MD simulation to calculate the structural deviations from 0 to 100 ns (Figures 4(a)–4(d)). Apparently, during the MD simulation process, the structural conformation and dynamic behavior of protein are based on the Root Mean Square Deviation (RMSD) [57]. The average potential energies of HSULF-2 and marmesin-HSULF-2 complex were computed to determine the interatomic forces, configured sample space, and generated the trajectory to know the equilibration status and the stability of the biological system at a specific time (Figure 4(a)). Similarly, the same procedure was extended to docked marmelosin-HSULF-2 complex to evaluate the equilibration and stability of the system (Figure 4(b)). The RMSD was recorded to rise from 0 ns and was steadily increased; the increasing trend can be observed from the plot throughout the 100 ns interval with multiple converging regions and very high correlation with protein-ligand; the RMSD values of the protein-ligand (marmesin-HSULF-2) complex were observed between 0.5 nm RMSD and 1.3 nm RMSD during the 100 ns except for a single spike at 90 ns. The mean RMSD value was recorded to be 0.8 nm. In the case of the marmelosin-HSULF-2 complex simulation (Figure 4(c)), the RMSD was recorded to increase from 0 ns (0.2 nm) steadily to reach 0.6 nm within 10 ns and the increasing trend with steady convergence with the protein backbone continued until 50 ns; a sudden rising trend was observed to reach 1.2 nm and the peak continued steadily in equilibrium throughout the 100 ns duration at 1.2 nm.

To investigate the average residual variations during the simulation process, a Root Mean Square Fluctuation (RMSF) study was carried out. The RMSF analysis was performed for 640 amino acids, including the ligand molecules (Figures 4(b) and 4(d)). The RMSF indicates the fluctuations mostly in the loop regions of the HSULF-2. It was significant in the identification and confirmation of minor fluctuations of atoms during the simulation process. The RMSF plot indicated minimal residual fluctuations in the regions of the protein-ligand interactions indicating higher stability. The H-bonds formed with the atoms of HSULF-2 and marmesin specify the stability and significant interactions between the protein and the ligand molecule (Figure 4(b)). Furthermore, in the HSULF-2-marmelosin complex's RMSF analysis, the interaction of atoms by H-bond formation indicated the stability and significant interaction between the HSULF-2-marmelosin complex (Figure 4(d)). It can be concluded that the marmelosin interaction with HSULF-2 is relatively weaker in comparison with the marmesin-HSULF-2 interaction.

## 4. Discussion

Herein, by using the gene-disease network analysis, we found that the oncoenzyme HSULF-2 plays an important role in several cancers and other noncommunicable diseases. Moreover, by using *in silico* approaches, we show the 3D structure of HSULF-2 and how phytochemicals found in the bael fruit extract, mainly marmesin and marmelosin, can interact with the enzyme's active site to inhibit its activity.

HSULF-2 is a heparan sulfatase-2 protein that exhibits 6-O-endo-sulfatase activity and that is differentially overexpressed on the surface of most cancer cell lines and is also found to be upregulated at the transcriptional level in ductal carcinoma *in situ* and invasive ductal carcinoma [29]. Moreover, our preceding study on various phytochemicals found in bael fruit, ginger, soya, and turmeric extracts exhibited the inhibition activity of HSULF-2 found in several cancerous cells and expressed at multiple stages of tumorigenesis [35]. Therefore, HSULF-2 can be ranked as a potential therapeutic target for the treatment of several cancers in which the enzyme plays a role in tumor progression [58].

A completely elucidated structure of HSULF-2 is unavailable in structure databases, so we modeled the structure based on homology modeling using the I-TASSER server, the model generated by the server was subjected to ModRefiner for further refining of the structure. Some regions exhibiting perturbations were omitted from the structure as they were falling towards the end regions of the structure and were not overlapping with the binding site or active site of the protein target. The predicted protein model exhibited 86.7% and 98.2% residues within the most favored and additionally allowed regions correspondingly in the Ramachandran plot predicted using PROCHECK. ProSA-web and Verify 3D predictions showed Z-score -5.48 and 69.36% quality factor, respectively; these analyses were evident to predict the low RMSD structure (62-64).

In the present *in silico* study, we have analyzed the binding ability of the phytocompounds from *A. marmelos* in the 3D structure of HSULF-2 active site. Traditionally, bioactive compounds of *A. marmelos* have been extensively applied in the therapy of many human diseases [4]. Earlier studies have reported the activity of the compounds marmesin and marmelosin, where marmelosin has exhibited antioxidant and anti-inflammatory properties in RAW264.7 cells [7]. Moreover, marmesin has antiangiogenesis properties in endothelial cells through the inactivation of VEGF-A-mediated cell signaling pathways [59, 60]. But reports on the inhibitory effect of HSULF-2 by marmesin and marmelosin are rare, but clearly shown in this study.

In the present study, we used computer-based drug design and virtual-screening methods to identify novel candidate-drug molecules (bael fruit's phytochemicals) based on the evaluation of the binding affinity and the binding modes with the ranked drug target (HSULF-2). Taking advantage of these computational tools, we have selected compounds found in the bael fruit extract and analyzed them for their binding potential and drug-likeness. In our molecular docking and molecular dynamics simulation study, we have used seven important nutraceutical components of bael fruits (i.e., marmesin, marmelosin, lupeol, ascorbic acid, psoralen, thiamine, and niacin) to investigate their binding in the 3D structure of the oncoprotein HSULF-2. Among them, and interestingly, marmesin and marmelosin exhibited maximum binding potential with HSULF-2. AutoDock Vina-based docking scores unveiled significant interactions of marmelosin with HSULF-2 indicating the strong inhibition of human HSULF-2 activity involved in various cancers and carcinomas. We anticipate that the pharmacological effect of marmesin and marmelosin, reported in the current study, maybe directional in concluding the drug activity with antiproliferative and anticancer properties subjected to further *in vitro* and *in vivo* studies.

Virtual screening has shown promising results in the computer-aided drug design and has been a significant step in identifying the most potential ligands in the preclinical phase of the drug screening steps due to the prediction potential and probability [61, 62]. The reliability of computational drug designing has increased in the current decade due to the advancement in the development of highly accurate prediction tools and methods. Taking advantage of these computational tools, we have selected compounds found in the bael fruit extract and analyzed them for their binding potential and drug-likeness. In our molecular docking and molecular dynamics simulation study, we have used seven important nutraceutical components of bael fruits (i.e., marmesin, marmelosin, lupeol, ascorbic acid, psoralen, thiamine, and niacin) to investigate their binding in the 3D structure of the oncoprotein HSULF-2. Among them, and interestingly, marmesin and marmelosin exhibited maximum binding potential with HSULF-2. AutoDock Vina-based docking scores unveiled significant interactions of marmelosin with HSULF-2 indicating the strong inhibition of human HSULF-2 activity involved in various cancers and carcinomas.

Inhibition of the HSULF-2 is advantageous in the inhibition of cancer progression as reported in our previous study [35]. The binding pocket is closely located (about 7 Å) to the active site CYS^88^ leading to *π*-*π*-stacking interactions with the active site residue. Our current study is consistent with that the drug candidate marmesin (PubChem CID: 334704) interaction with HSULF-2 is stabilized by multiple H-bonds with ARG^538^ (3.36 Å), LYS^526^ (3.73 Å), ARG^406^ (2.87 Å), GLU^543^ (3.76 Å), and ASP^661^ (3.84 Å), and hydrophobic interactions with LYS^403^, VAL^544^, ILE^542^, SER^539^, LYS^528^, CYS^455^, MET^405^, LYS^404^, LYS^526^, and LYS^525^. The interacting-binding pocket residues LYS^526^, ARG^538^, and GLU^543^ with marmesin are closely located with the mutated residues ASP573 and TYR^531^.

Furthermore, the second candidate molecule marmelosin (PubChem CID: 10212) interaction was further stabilized with multiple H-bonds with CYS^455^ (2.57 Å), MET^405^ (5.29 Å, 5.74 Å), LYS^521^ (3.25 Å, 3.50 Å), LYS^403^ (3.59 Å), and GLN^612^ (3.74 Å, 3.55 Å), and hydrophobic interactions with ARG^519^, SER^411^, LYS^525^, LYS^526^, LEU^615^, VAL^544^, LEU^522^, and ARG^518^. Like marmesin, marmelosin exhibits significant interactions with the binding pocket residues leading to the inhibition of the HSULF-2 supported by hydrophobic interactions. The H-bonds LYS^521^ (3.25 Å, 3.50 Å) and GLN^612^ (3.74 Å, 3.55 Å) were found to be closely located with mutated residues.

The family of sulfatase enzymes catalyses the hydrolysis of sulfate bonds in various substrates, including sulfated proteoglycans, steroids, and esters. Among the seventeen human sulfatases identified, many are linked with genetic disorders leading to reduced or loss of function of the respective enzymes [16]. The increased homology among the protein sequences has led to high conservation in the tertiary structure and the active site. The conserved catalytic active residue structures hint at common catalytic mechanisms adopted by these enzymes. Structural analysis of catalytically active sulfatases reveals that catalytically significant cysteine residue is conserved in all known sulfatases and is posttranslationally modified into multiple states [16]. In the docking study, as mentioned above, the results indicate the significant interaction of the prioritized ligands interacting closely with catalytic CYS^455^ residue by forming *π*-alkyl and hydrogen bonds. Furthermore, these interactions are facilitated by alkyl, *π*-alkyl, *π*-sulfur, and hydrophobic interactions. These interactions indicate the therapeutic significance of inhibition of the sulfatase activity.

The protein-ligand affinity score is predicted by an empirical-scoring function influenced by the X-score function adopted by AutoDock Vina (ADV) [50]. The empirical-scoring function is more oriented towards machine learning than available physics-based functions. ADV has implemented a simpler scoring function with fewer parameters, which helps readily identify the conventional terms in force fields. ADV summates the individual terms, where each term is related to the important energetic factor in protein-ligand interaction [50]. Depending upon the parameters available for individual functions, the parameters can be altered to enhance the prediction of binding interactions and affinity. Furthermore, ADV sums up the contributions of individual terms. Each of these unique terms corresponds to a significant energetic factor in molecular interaction. Based on the available parameters in individual functions, they can be modified to improvise the binding affinity prediction. The binding energy is estimated based on the sum of the distance-dependent atomic interactions. ADV uses various scoring functions for including/excluding several interactions, such as like hydrogen bonds, electrostatic interactions, hydrophobic interactions, and Gaussian steric attractions. Compared with AutoDock 4 (AD4), ADV-scoring function are altered, as they represent the general functional form of the confirmation-dependent, as stated in the original publication [50]; it includes van der Waals-like potential and it does not possess electrostatics and solvation term. Additionally, the hydrophobic term, omnidirectional hydrogen-bond term, and penalty for conformational entropy are adopted [63]. For the optimization procedure, ADV explores stochastic global optimization approaches, like genetic algorithm and simulated annealing, in combination with different local optimization steps and customized “tricks” to increase the robustness of the optimization procedure. Overall, it led to the better performance and speed of the ADV over AD4.

Molecular dynamics simulation has enhanced the computational prediction ability based on Newtonian physics [64–67] to analyze the dynamics of the biomolecular interactions. Molecular dynamics simulation is a standard procedure followed to understand and analyze the structural dynamics and variation in the stability of the protein target after ligand binding [68, 69]. Many of the earlier published reports suggest the high concordance between the experimental and computational-based studies.

The molecular dynamics results showed the variation in the structural stability and confirmation of HSULF-2 upon binding of marmesin and marmelosin candidates. However, from Figure 4, we show that the RMSD of APO and the ligand-bound protein structure were stabilized. The RMSF suggests that the fluctuations were reduced leading to a change in confirmation after the binding of the ligands. Marmesin showed better interactions leading to higher conformational changes in comparison with marmelosin [70–72].

Furthermore, by MD simulation time-dependent and thermodynamics properties, conformational changes can be analyzed. It is achieved by precisely selecting the number of frames from MD simulations to justify the theoretical outcomes without losing the appropriate data from the simulation experiment. Novel methods have been adopted based on statistical mechanics (SMs), principal component analysis (PCA), and wavelet analysis (WA) for specifically selecting MD conformations [63]. The macroscopic properties are understood with the application of statistical mechanics, which provides a detailed background of mathematical expressions relating to the atomic and molecular distributions and the motions in macroscopic observables (free energies, heat capacity, and pressure). With the knowledge of microscopic data, the macroscopic observables can be extracted, and predictions including binding free energy (e.g., phytochemicals), interaction mechanisms, and energetic favorability of conformational alterations (e.g., HSULF-2) can be made. Specificities of thermodynamics and kinetics of biomolecular structure can be analyzed in various applications of interactions, properties, and dynamics of small molecules and macromolecules [73]. It is also advantageous in molecular-designing studies (protein/drug/antibody), structure elucidation, and refinement studies (NMR and X-ray) [74, 75].

The PCA method significantly reduces the dimensionality of the MD simulation data set. It gives the linear combination of the variables by providing the best-fit line throughout the complete dataset. Based on it, the highest amount of variance in an arbitrary dataset can be drawn. Subsequently, principal components are linked to the remaining residual variance after filtering the important principal components. In the trajectory analysis, the challenge lies in separating the functionally significant motions with random thermal fluxes in a protein target. In extracting important, extensive, correlated motions in MD simulation, PCA is considered a potential method. The principal components involved in the highest alteration can be adopted onto the protein target, individually or as a set, and these principal components act as the orthogonal basis set for atomic coordinates in individual trajectories. The remaining variations, including minor thermal variations, can be filtered. Overall, PCA enables the visualization and orientation of the major fluctuations relevant to the biological significance [76, 77].

Wavelet transform (WT) analysis is recommended as a molecular dynamics analysis tool based on its application in signal and image processing [78]. WT is a popular tool in molecular biology helpful in analysing the sequences and protein structures, including the rare occurrences, alterations that are localized at a specific time scale, and frequency molecular dynamics simulations of HSULF-2-phytochemical interactions. Additionally, it is an effective method for separating phenomena of the different time courses. This way, it has reduced valuable noise by filtering the high-frequency variations without disturbing the high-frequency phenomena. Recently, the continuous wavelet transform (CWT) analysis has been suggested to be an effective method in MD simulations [78]. Similar to the Fourier transform, WT provides information on the signal's frequency domain. Additionally, WT is useful in extracting instantaneous data on how a specific frequency is localized at a particular time frame. Subsequently, wavelets can be employed to obtain crucial data about different modes of a specific signal without data loss, the frequency of the occurrences of the modes, and how transformative the modes are. These above-mentioned methods may be considered as a theoretical strategy to selectively choose the promising configurations from the MD simulation to determine the theoretical accuracy and reduce the number of frames of MD simulations.

## 5. Conclusions

The molecular docking and molecular dynamics calculations and the computational data obtained from the results suggest that the pharmacological effect of marmesin and marmelosin, reported in the current study, may be directional in concluding the drug activity with antiproliferative and anticancer properties bound to further *in vitro* and *in vivo* studies. Marmesin and marmelosin interact at the active site of HSULF-2 causing its inhibition. These phytochemicals found in the bael fruit extract can serve as structural platforms for the design of novel drugs for the treatment of cancers in which the activity of HSULF-2 is critical for tumor progression.

## Figures and Tables

**Table 1:** Chemical properties of selected phytochemicals found in bael fruit's extracts.

Properties	Marmesin	Marmelosin	Lupeol	Ascorbic acid	Niacin	Psoralen	Thiamine
mol. Weight	246.26 g/mol	270.28 g/mol	426.72 g/mol	176.12 g/mol	123.11 g/mol	186.16 g/mol	265.35 g/mol
H-bond Acceptors	4	4	1	6	3	3	3
H-bond donors	1	0	1	4	1	0	2
TPSA score (Å^2^)	59.67	52.58	20.23	107.22	50.19	43.35 Å	104.15
Bioavailability	0.55	0.55	0.55	0.56	0.85	0.55	0.55
Log *P*	2.55	3.05	4.72	-0.52	0.86	2.01	-1.6
Log S (ESOL)	-2.92	-4.00	-8.64	-0.2	-1.26	-2.73	-2.32
Log S (Ali)	-2.79	-4.29	-10.22	-0.82	-0.98	-2.19	-2.8
GI absorbtion	High	High	Low	High	High	High	High
BBB permeate	Yes	Yes	No	No	Yes	Yes	No
Lipinski	Yes 0 violation	Yes 0 violation	Yes 1 violation MLOGP > 4015	Yes 0 violation	Yes 0 violation	Yes 0 violation	Yes 0 violation
PAINS	0 alert	0 alert	0 alert	0 alert	0 alert	0 alert	0 alert

**Table 2:** Binding energy scores (AutoDock scores) of selected phytochemicals found in bael fruit extracts with HSULF-2.

Molecule	Binding energy (kcal/mol)	RMSD/ub = 0	RMSD/lb = 0	Hydrogen bonds, pi-cation, pi-anion, and pi-sulfur	Hydrophobic interactions, alkyl/pi-alkyl
Marmesin	-8.5	**0**	**0**	ARG-538 (3.36 Å), LYS-526 (3.73 Å), ARG-406 (2.87 Å), GLU-543 (3.76 Å), and ASP-661 (3.84 Å)	LYS-403, VAL-544, ILE-542, SER-539, LYS-528, CYS-455, MET-405, LYS-404, LYS-526, and LYS-525
Marmelosin	-7.7	**0**	**0**	CYS-455 (2.57 Å), MET-405 (5.29 Å, 5.74 Å), LYS-521 (3.25 Å, 3.50 Å), LYS-403 (3.59 Å), and GLN-612 (3.74 Å, 3.55 Å)	ARG-519, SER-411, LYS-525, LYS-526, LEU-615, VAL-544, LEU-522, and ARG-518
Lupeol	-7.0	**0**	**0**	TYR-531 (2.27 Å), ASN-103 (3.12 Å), TRP-118 (2.88 Å), and HIS-102 (2.91 Å)	ARG-533, ILE-377, SER-534, SER-536, ASP-376, and ARG-125
Ascorbic acid	-4.9	**0**	**0**	ARG-416 (2.12 Å), VAL-101 (3.06 Å), ASN-105 (3.27 Å), and GLU-450 (2. 76 Å)	TYR-107, GLY-417, TYR-451, GLU-415, and THR-453
Psoralen	-6.9	**0**	**0**	ARG-406 (3.36 Å), GLU-543 (3.49 Å, 3.90 Å), and LYS-526 (3.07 Å)	MET-405, LYS-525, LYS-526, VAL-544, and ARG-538
Thiamine	-6.6	**0**	**0**	ALA-449 (2.83 Å), CYS-446 (2.94 Å), and GLU-415 (2.39 Å)	HIS-102, CYS-446, ARG-416, GLU-450, and TYR-107
Niacin	-5.2	**0**	**0**	VAL-101 (2.48 Å), ASN-105 (3.47 Å), TYR-451 (2.88 Å), and ARG-416 (2.57 Å)	TYR-107, THR-453, GLU-415, and GLU-450

## Data Availability

The authors declare that all the relevant data supporting the findings of this study are available within the paper and its supplementary information and from the corresponding authors upon reasonable request. The code is available from the corresponding authors upon reasonable request.

## Supplementary Materials

Supplementary Materials Supplementary Figure 1: involvement of HSULF-2 in human diseases. (A) Disease-gene network annotation with HSULF-2 with distance and threshold value 4.0. (B) STRING-based P-P interaction network of the proteins with high correlation. Supplementary Table 1: the annotated diseases are listed from DisGeNET, Diseases, DOAF, and GOBP databases for *hsulf-2* gene. Cancers and carcinoma are major among the list of annotated diseases.
