# Supplementary material for: Marmesin and Marmelosin Interact with the Heparan Sulfatase-2 Active Site: Potential Mechanism for Phytochemicals from Bael Fruit Extract as Antitumor Therapeutics
Source: Oxid Med Cell Longev. 2023 Jan 5;2023:9982194. doi: 10.1155/2023/9982194 (PMC9836799; doi:10.1155/2023/9982194)
Supplement: Supplementary Materials — Supplementary Figure 1: involvement of HSULF-2 in human diseases. (A) Disease-gene network annotation with HSULF-2 with distance and threshold value 4.0. (B) STRING-based P-P interaction network of the proteins with high correlation. Supplementary Table 1: the annotated diseases are listed from DisGeNET, Diseases, DOAF, and GOBP databases for hsulf-2 gene. Cancers and carcinoma are major among the list of annotated diseases. [file 9982194.f1.zip › Suppl Table 1.docx]

**Supplementary Table 1. The annotated diseases are listed from DisGeNET, DISEASES, DOAF and GOBP databases for *hsulf-2* gene.** The cancers and carcinoma are major among the list of annotated diseases.

| **DisGeNET** | **DISEASES** | **DOAF** | **GOBP** |
| --- | --- | --- | --- |
| - Endometrioma - Endometriosis - Malignant neoplasm of breast | - Aleutian mink disease - Breast disease - Cancer - Carcinoma - Connective tissue disease - Hematopoietic system disease - Liver disease - Lung disease - Pancreas disease | - cancer - carcinoma - hepatocellular carcinoma - malignant neoplasm of breast - myeloma - osteoarthritis | - bone development - cartilage development - chondrocyte development - embryonic skeletal system development - glial cell-derived neurotrophic factor receptor signaling pathway - glomerular basement membrane development - glomerular filtration - innervation - kidney development - liver regeneration - negative regulation of fibroblast growth factor receptor signaling pathway - positive regulation of Wnt signaling pathway - positive regulation of canonical Wnt signaling pathway - positive regulation of vascular endothelial growth factor production - response to wounding |
