# Supplementary figures and images for: Marmesin and Marmelosin Interact with the Heparan Sulfatase-2 Active Site: Potential Mechanism for Phytochemicals from Bael Fruit Extract as Antitumor Therapeutics
Source: Oxid Med Cell Longev. 2023 Jan 5;2023:9982194. doi: 10.1155/2023/9982194 (PMC9836799; doi:10.1155/2023/9982194)

## Slide 1
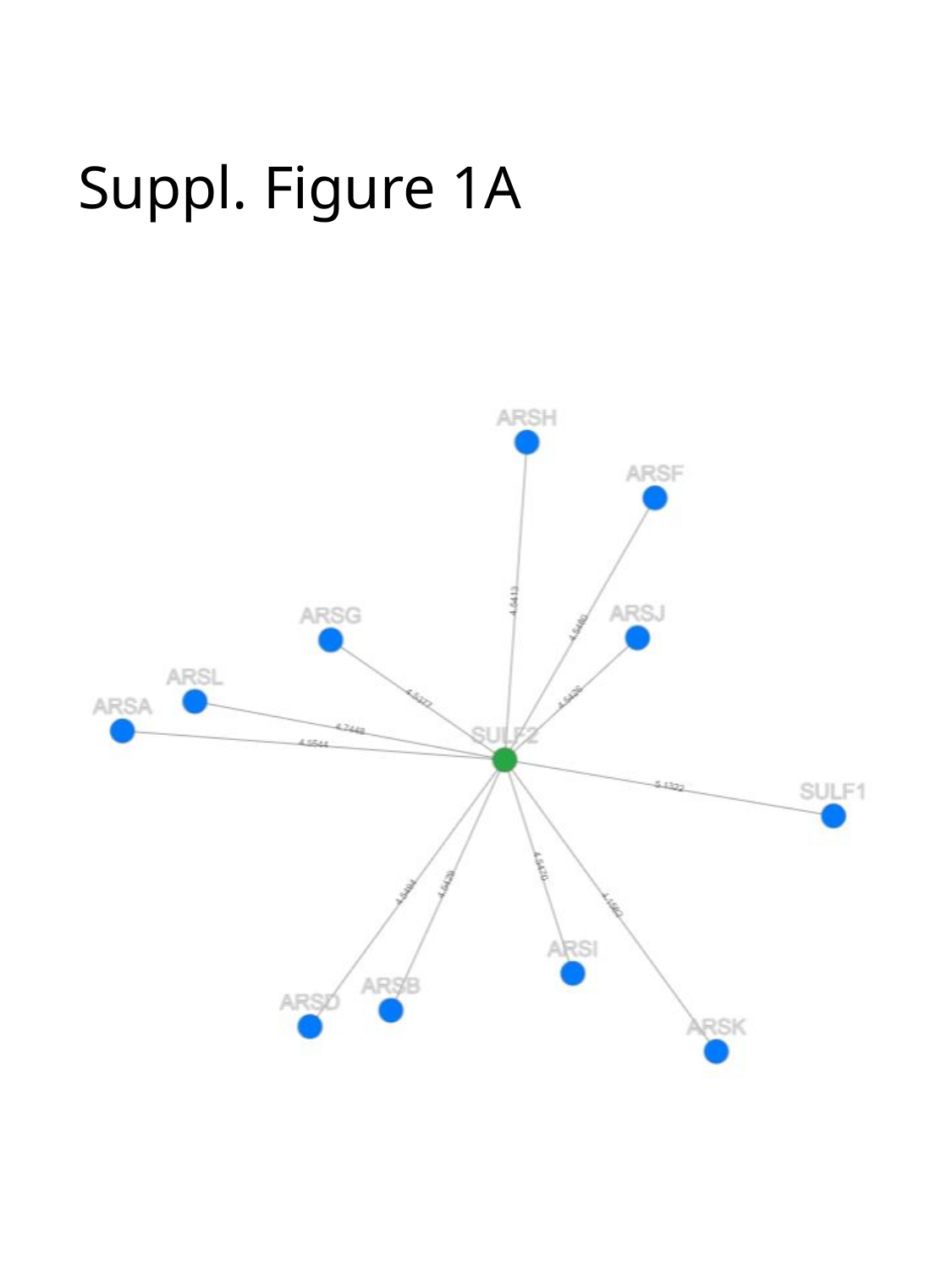

# Suppl. Figure 1A

Supplement: Supplementary Materials — Supplementary Figure 1: involvement of HSULF-2 in human diseases. (A) Disease-gene network annotation with HSULF-2 with distance and threshold value 4.0. (B) STRING-based P-P interaction network of the proteins with high correlation. Supplementary Table 1: the annotated diseases are listed from DisGeNET, Diseases, DOAF, and GOBP databases for hsulf-2 gene. Cancers and carcinoma are major among the list of annotated diseases. [file 9982194.f1.zip › SUPPL. FIGURE 1A.pptx]

## Slide 1
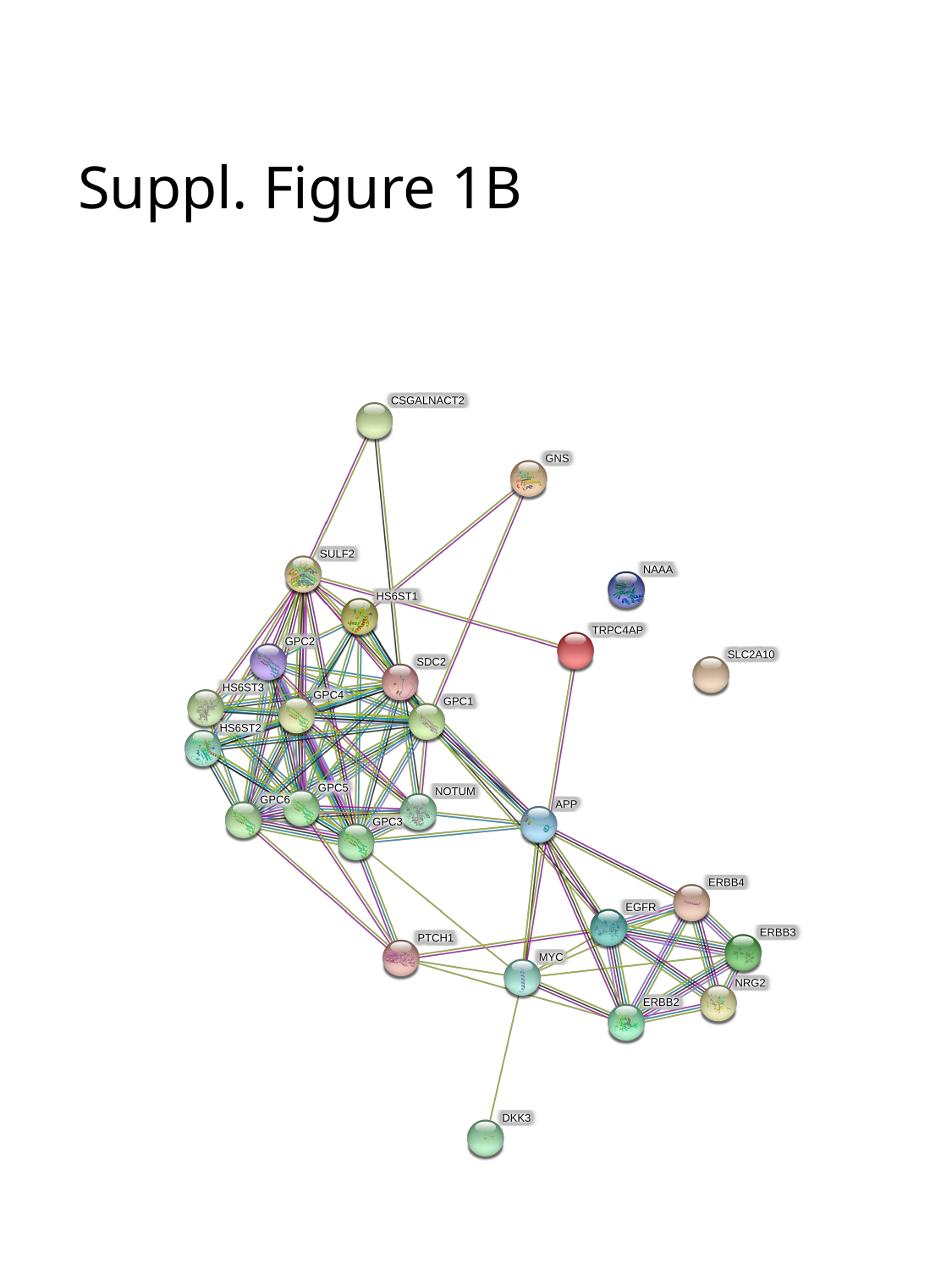

# Suppl. Figure 1B

Supplement: Supplementary Materials — Supplementary Figure 1: involvement of HSULF-2 in human diseases. (A) Disease-gene network annotation with HSULF-2 with distance and threshold value 4.0. (B) STRING-based P-P interaction network of the proteins with high correlation. Supplementary Table 1: the annotated diseases are listed from DisGeNET, Diseases, DOAF, and GOBP databases for hsulf-2 gene. Cancers and carcinoma are major among the list of annotated diseases. [file 9982194.f1.zip › SUPPL. FIGURE 1B.pptx]
